# Which classification system could empower the understanding of caesarean section rates in Greece? A review of systematic reviews

**DOI:** 10.18332/ejm/147993

**Published:** 2022-06-16

**Authors:** Paraskevi Giaxi, Kleanthi Gourounti, Victoria G. Vivilaki, Katerina Lykeridoy

**Affiliations:** 1 Department of Midwifery, School of Health and Care Sciences, University of West Attica, Athens, Greece

**Keywords:** caesarean section, classification, taxonomy, optimal rate of caesarean section, midwifery, empowerment, psychometric properties, exploratory factor analysis, assessment tool

## Abstract

**INTRODUCTION:**

Worldwide there is an alarming increase in the caesarean delivery rate which has become a controversial topic. However, the reasons for this tendency are not clear. For example, in Greece alone, rates increased by almost 50% from 1983 to 1996. In order to better understand the causes of this phenomenon, we need to examine closely what groups of women undergo caesarean section (CS). To achieve this, it is essential to use a system that will enable us to monitor and compare caesarean delivery rates. Such a classification system should be easily adopted by obstetricians, midwives, and public health services.

**METHODS:**

A review search of electronic databases concerning medical care was held from December 2020 to January 2021 in order to find systematic reviews which describe either theoretical or practical CS classification systems.

**RESULTS:**

The most common classification systems fall into three main categories based on indication, urgency and maternal-based characteristics. According to users the highest rated classification system was women-based classifications in general. In particular the Robson Ten Group Classification System was considered to be the most valid to meet current local and international standards. The Robson classification system is praised for its robustness, simplicity, flexibility, and reproducibility.

**CONCLUSIONS:**

The right implementation of the Robson Ten Group Classification System can facilitate an in-depth analysis of the main groups that increase CS rates and can be used to both review and monitor delivery practices both in Greece and abroad.

## INTRODUCTION

Worldwide there is an alarming increase in caesarean delivery rates but the reasons for this phenomenon are not fully understood^1,2^. The World Health Organization (WHO) together with the International Federation of Obstetrics and Gynecology (FIGO) have expressed their concern at this steady increase, considering this international trend an ‘epidemic’^3,4^. Since 1985, the World Health Organization (WHO) has considered that there should be no justification for CS rates over 10–15% , for whatever region. Since then, the validity of this threshold has been questioned and the international community has increasingly referenced the need to revise the 1985 recommended rate^4^. According to the latest research, this threshold has been raised to around 19%, with no significant change on maternal and neonatal mortality^5^. Strictly speaking, worldwide caesarean section rates have risen from about 6% in 1990 to 19% in 2014^6^. In the northern part of Europe, rates are still under 20% while in the southeastern part of Europe, China and South America they have climbed to or above 50% of total births^5^. In northern African countries the rates have risen from 5% to 28%. In Egypt this rate has reached 50%^1^. Caesarean section rates have remained very low in rural areas of low-income and middle-income countries. However, there has been an increase in urban areas. This can be easily understood, as there is a visible lack of access to medical services in certain regions while there is an overuse of interventions in other parts of the world or in other areas of the same country^7^. Those large variations in caesarean birth rates have become a major, controversial public health concern^8,9^ and rising CS rates give rise to international debates owing to possible perinatal risks associated with this rise, limited access and issues related to cost^4,10-13^. Consequences include direct and indirect perinatal morbidity and mortality caused by bleeding, aesthetic and urological complications, thromboembolism and infection, respiratory neonatal problems^14^. Moreover, the increase in CS rates negatively affects future pregnancies as there is a related rise in preterm birth and abnormalities of the placenta, which may lead to excessive vaginal bleeding and as a result to hysterectomy^15^.

In Greece, the situation is even worse, over half of the births occur by CS, making it one of the countries with the highest CS rates in the world. In Greece, rates increased by almost 50% from 1983 to 1996^16^. In their 2005 study, Mosialos et al.^17^ reported a 41.6% CS rate in two public hospitals and a 53% CS rate in a private hospital. In the same study, authors investigated the factors that increased the likelihood of undergoing CS. Results of this study led to the conclusion that obstetricians tend to perform a CS for financial and convenience reasons.

In order to better understand the causes of this phenomenon, it is first imperative to examine closely the groups of women who are undergoing CS. This requires using a classification system which could best monitor and compare CS rates in a uniform, dependable, and consistent manner. Such a system should be ‘simple, accountable, clinically relevant, verifiable and replicable’ and globally applicable and useful for healthcare providers and public health services^18^.

### Aim

The aim of this review is to identify the most reliable classification system which is appropriate, and that could monitor, assess and compare the ever-increasing caesarean section rates.

## METHODS

A review search of electronic databases concerning medical care (PubMed, Medline, Cochrane, CINAHL, Scopus, WHO) was conducted from December 2020 to January 2021 in order to detect systematic reviews which presented either a theoretical or a practical caesarean delivery classification system, published in English from inception to 2021. For the systematic search of the databases the MeSH terms, PICO and PICO management elements were used, depending on the database, with the following search terms: caesarean section, classification or taxonomy, and optimal rate of caesarean section. Additional methods of searching included a search of the reference list of articles selected from the primary search, in order to identify studies which had not surfaced from the initial search.

### Inclusion and exclusion criteria

The included studies were systematic reviews, published from inception to January 2021, in the English language. The included studies assessed any type of classification systems for CS which gave a clear description. The review included studies based on the use of a classification system for CS in various groups of low-risk women, regardless of their perinatal, medical or other demographic characteristics.

### Study screening and study selection

**Figure 1:**
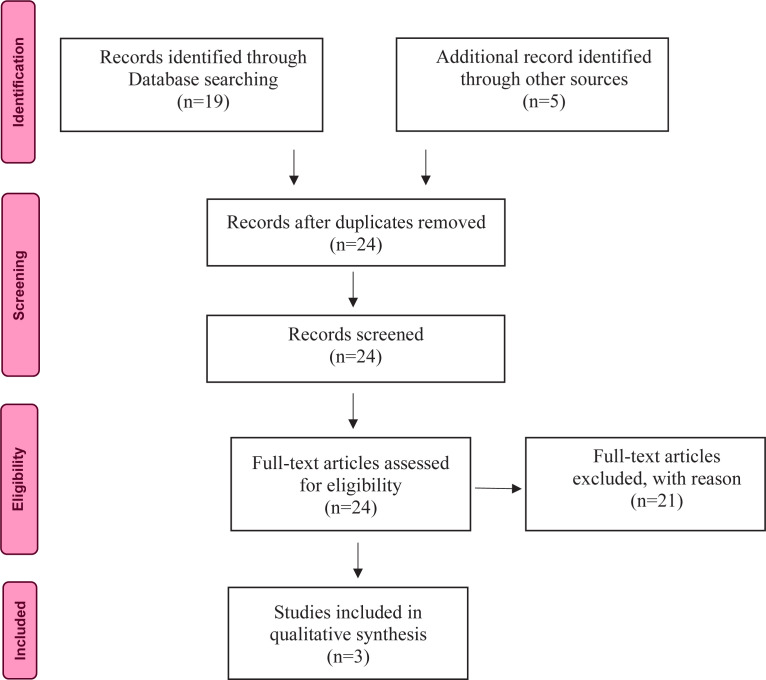
PRISMA flowchart

Data collection and analysis was conducted by two authors simultaneously. The initial search generated 24 titles from the database systematic search. The study selection flow diagram is shown in Figure 1. The screening and selection of studies was performed in January 2021 using the ROBIS checklist for umbrella reviews. Titles and abstracts were assessed for relevance to the review objective. After the evaluation of the titles and abstracts, 21 were omitted for the following reasons: 17 studies were excluded because the research method was not a systematic review of the literature and 4 studies observed different cases of interest. For the remaining 3 studies, only relevant data were included (Table 1).

**Table 1:** Characteristics of included studies

*Study*	*Country*	*Study design*	*Outcome investigated*	*Results*
Torloni et al.^19^ 2011	Argentina	Systematic review	Identify the main CS classifications used worldwide	Women-based classifications in general and Robson classification in particular would be the best position to fulfill current international needs.
Betrán et al.^21^ 2014	United States of America	Systematic review	To gather the experience of users related to pros and cons of the adoption, implementation and interpretation of the Robson classification	The use of Robson classification is increasing rapidly and spontaneously worldwide. It is easy to implement and interpret. Modifications could be useful.
Longo et al.^22^ 2020	Italy	Systematic review	Contribute to the contextual understanding of the increasing number of CSs being performed in Italy.	Mitigating the high rates of CSs will require a synergistic-stakeholder intervention. Although they did not apply any classification system in their research, they suggested the implementation of Robson for a better insight the causes of growing CS rates and for the development of constrictive interventions.

## RESULTS

In a 2011 WHO systematic review several classification methods were examined. These classifications can be divided into three main categories: 1) indication-based, 2) urgency-based, and 3) women-based. Of these, the indication-based system was the most commonly used. The main question answered by this type of classification is ‘why’ the CS is performed. These data are recorded routinely in all maternity hospitals therefore it is easy to implement. However, the weakness of this taxonomy was that women giving birth with CS could fall into more than one category (could be >1 primary indication). Another weakness is that there are no uniform definitions for frequent indications (e.g. fetal distress, failure to progress, dystocia). Moreover, the indications could only be reviewed after the performance of the CS, rendering implementation difficult and not very useful for the reformation of clinical practice. Classifications according to the degree of urgency for caesarean births were easy to interpret and implement owing to the limited number of the proposed categories. Thus, providing valuable information regarding ‘when’ the CS is performed. The main strength of this type of classification is that it could improve the communication among health professionals and in this way have a positive impact on maternal– neonatal outcomes. However, in this type of group similar problems were noted with indication-based classifications. Classifications according to the level of urgency do not provide accurate definitions for each of the categories. They have poor reproducibility unless clearer definitions are provided and medical staff are better trained. Also, they possess limited value for health policy makers, epidemiologists and public health authorities. Women-based classifications essentially inform us who is to undergo CS, according to maternal and pregnancy characteristics and are considered as the most advanced approach. These classification systems allow prospective categorization into mutually exclusive, completely inclusive categories and are highly dependable. Also, they have been performed in various countries and in large parts of the population. Robson Ten Group Classification System and the Denk 8 group classification system were given the highest overall ratings. Specifically, they have been characterized as flexible enough to been employed in different clinical settings. One of the advantages is that they offer the opportunity to implement modifications in clinical practices in order to manage CS rates. The only drawback of women-based classifications is that they fail to mention why (indications) or when (degree of urgency) the CS was conducted. According to a 2011 systematic review by Torloni et al.^19^, the most highly rated classification system was women-based in general and Robson classification system in particular. The latter is acknowledged to be the most appropriate to meet current regional and international standards^19^. According to the Robson system all deliveries can be categorized into one of the ten groups based on five parameters: obstetric history, (parity and previous caesarean section), onset of labor (spontaneous, induced, or caesarean section before onset of labor), fetal presentation or lie (cephalic, breech, or transverse), number of neonates and gestational age^20^. The classification categorizes all women admitted for delivery into one of ten groups, which are mutually exclusive and totally inclusive. In other words, adopted based on a few basic obstetric parameters. All women admitted for delivery in any hospital can be classified into only one of the ten groups and no woman is excluded^20^.

In 2015, Betran et al.^10^ carried out a systematic review in order to synthesize the experience of the users of the Robson classification system regarding the advantages and disadvantages of the adopting, implementing and interpreting of this particular classification system, together with their modifications or recommendations regarding this classification. According to the author, the Robson classification system is praised for its robustness, simplicity, flexibility and reproducibility and the fact that it categorizes women prospectively allowing the adoption and assessment of interventions directed at specific groups. According to the users, the classification is capable of overcoming the main weaknesses of those systems that are not mutually exclusive and have low reproducibility for frequent reasons such as dystocia or fetal distress. In a systematic review^21^ of the Robson classification which examines its use in over 33 million women from 31 countries, out of 58 studies, 34 studies produce data according to the Robson ten groups without subdivisions and 18 studies provide their data according to subgroups or even adding new groups. Moreover, they report that the classification is clinically applicable and it can be employed as a tool to lower CS rates by itself. They concluded that several authors proposed relevant modifications that could pave the way for their implementation by medical facilities worldwide and suggest that assessing maternal and fetal outcomes in relation to CS rates would be the next critical step towards establishing an optimal caesarean section rate.

A 2020 systematic review by Longo et al.^22^, examined the increase in the number of CSs performed in Italy from the perspective of a healthcare system. They reported that CS rates in Italy are influenced by complex interrelationships, by several involved and specific circumstances and factors such as the over-medicalization of delivery, legal and social issues, variations in practice and policies across healthcare institutions and national nations, and women’s point of view regarding pregnancy and childbirth. Authors recommended that the high CS rate in Italy justifies a close monitoring of epidemiological data and hospital practices relevant to CS. Although they did not apply any classification system in their research, they suggested the implementation of Robson classification, especially in less centralized hospitals and private units, for a better insight into the causes of the growing CS rates and for the development of interventions to safely curb this phenomenon.

## DISCUSSION

Our findings show that worldwide, there is a rapid increase in the use of the Robson classification. Unlike previous classifications based on indication for CS, the Robson classification is a comprehensive system including all women, regardless of the mode of birth or circumstances (e.g. maternity hospital or area). WHO proposes the Robson classification system ‘as a global standard for assessing, monitoring and comparing caesarean section rates within healthcare facilities over time, and between facilities’^4^. According to the WHO experts, the use of the Robson classification will enable hospitals to identify and analyze the groups of women which influence to a greater or lesser extent the overall CS rates. Robson classification could compare delivery practices in these groups of women with other hospitals which have more covetable results and consider changes accordingly. Moreover, Robson classification will help healthcare facilities to evaluate the effectiveness of interventions or the strategies employed at optimizing the use of CS. Also, it will be able to evaluate the quality of clinical care and management of clinical practices by analyzing outcomes within groups of women. Furthermore, it could appraise the validity of the data collected and alter the staff about their importance, interpretation and use^20^.

According to our review, many authors who used the Robson classification proposed subdivisions in the basic 10 groups. The aim of proposed subdivisions is to raise consistency and homogeneity of the groups by categorizing women within groups according to certain relevant criteria. In 2012, the Society of Obstetrics and Gynecology, Canada (SOGC), published the modified Robson criteria (Table 2). This modification includes the following subdivisions: 1) after spontaneous onset of labor, 2) after induction of labor, and 3) before labor^23,24^. According to WHO, sub-classifications are particularly appropriate when deciding the kind of clinical interventions that will be implemented in specific subgroups. WHO specifically emphasizes that all subdivisions must be analyzed in relation to one another, and not by themselves, in order to arrive at a valid outcome^20^. According to WHO, Table 3 presents the most frequent subcategories.

**Table 2:** The modified Robson classification

1.	Nullipara, singleton cephalic, ≥37 weeks, spontaneous labor
2a.	Nullipara, singleton cephalic, ≥37 weeks (induced)
2b.	Nullipara, singleton cephalic, ≥37 weeks (Caesarean section before labor)
3.	Multipara, singleton cephalic, ≥37 weeks, spontaneous labor
4a.	Multipara, singleton cephalic, ≥37 weeks (induced)
4b.	Multipara, singleton cephalic, ≥37 weeks (Caesarean section before labor)
5a.	Previous Caesarean section, singleton cephalic, ≥37 weeks (spontaneous labor)
5b.	Previous Caesarean section, singleton cephalic, ≥37 weeks (induced)
5c.	Previous Caesarean section, singleton cephalic, ≥37 weeks (Caesarean section before labor)
6a.	All nulliparous breeches (spontaneous labor)
6b.	All nulliparous breeches (induced)
6c.	All nulliparous breeches (Caesarean section before labor)
7a.	All multiparous breeches (including previous Caesarean section) (spontaneous labor)
7b.	All multiparous breeches (including previous Caesarean section) (induced)
7c.	All multiparous breeches (including previous Caesarean section) (Caesarean section before labor)
8a.	All multiple pregnancies (including previous Caesarean section) (spontaneous labor)
8b.	All multiple pregnancies (including previous Caesarean section) (induced)
8c.	All multiple pregnancies (including previous Caesarean section) (Caesarean section before labor)
9a.	All abnormal lies (including previous Caesarean section but excluding breech) (spontaneous labor)
9b.	All abnormal lies (including previous Caesarean section but excluding breech) (induced)
9c.	All abnormal lies (including previous Caesarean section but excluding breech) (Caesarean section before labor)
10a.	All singleton cephalic, ≤36 weeks (including previous Caesarean section) (spontaneous labor)
10b.	All singleton cephalic, ≤36 weeks (including previous Caesarean section) (induced)
10c.	All singleton cephalic, ≤36 weeks (including previous Caesarean section) (Caesarean section before labor)

**Table 3:** The 10 groups of the Robson classification with subdivisions by WHO

1.	Nulliparous women with a single cephalic pregnancy, ≥37 weeks gestation in spontaneous labor
2a.	Nulliparous women with a single cephalic pregnancy, ≥37 weeks gestation who had labor induced or were delivered by CS before labor (labor induced)
2b.	Nulliparous women with a single cephalic pregnancy, ≥37 weeks gestation who had labor induced or were delivered by CS before labor (Pre-labor CS)
3.	Multiparous women without a previous CS, with a single cephalic pregnancy, ≥37 weeks gestation in spontaneous labor
4a.	Multiparous women without a previous CS, with a single cephalic pregnancy, ≥37 weeks gestation who had labor induced or were delivered by CS before labor (labor induced)
4b.	Multiparous women without a previous CS, with a single cephalic pregnancy, ≥37 weeks gestation who had labor induced or were delivered by CS before labor (Pre-labor CS)
5a.	All multiparous women with at least one previous CS, with a single cephalic pregnancy, ≥37 weeks gestation (with one previous CS)
5b.	All multiparous women with at least one previous CS, with a single cephalic pregnancy, ≥37 weeks gestation (with two or more previous CSs)
6.	All nulliparous women with a single breech pregnancy
7.	All multiparous women with a single breech pregnancy including women with previous CS(s)
8.	All women with multiple pregnancies including women with previous CS(s)
9.	All women with a single pregnancy with a transverse or oblique lie, including women with previous CS(s)
10.	All women with a single cephalic pregnancy <37 weeks gestation, including women with previous CS(s)

## CONCLUSIONS

The right implementation of the Robson classification can contribute to a better understanding of the main groups that increase caesarean birth rates in Greece and to use it as a useful tool to both audit and monitor our practice in our country and/or between countries. Itself, the Robson classification could be used as a tool intervention for the monitoring and reduction of CS rates. Also, Robson classification could help individual populations with the impact of caesarean section on both maternal and neonatal morbidity and mortality rates. Moreover, it could help customize our practice for optimum perinatal health. However, the Robson system does not take account for other maternal and perinatal aspects such as: maternal age, pre-existing conditions, body mass index (BMI), type of labor, oxytocin, indications for caesarean section or non-clinical variables that influence the CS rate, concluding that more variables could be included and analyzed regarding each and every group of Robson classification in the Greek population.

## Data Availability

Data sharing is not applicable to this article as no new data were created.
